# An incisional hernia containing a gangrenous gallbladder: a case report and review of the literature

**DOI:** 10.1093/jscr/rjac536

**Published:** 2022-12-07

**Authors:** Timbre Backen, W Tyler Crawley, Travis Bouchard, Glenda Quan

**Affiliations:** General Surgery Residency Program, Swedish Medical Center, Englewood, CO, USA; General Surgery Residency Program, Swedish Medical Center, Englewood, CO, USA; General Surgery Residency Program, Swedish Medical Center, Englewood, CO, USA; Department of Graduate Medical Education, Swedish Medical Center, Englewood, CO, USA

## Abstract

We present a 76-year-old male who presented to the emergency department with 24 hours of sudden onset, severe abdominal pain. Physical exam and laboratory analysis indicated acute cholecystitis, and a CT scan demonstrated a ventral hernia containing an inflamed gallbladder. This patient was managed operatively with an open cholecystectomy. The ventral hernia was not repaired at the index operation in the setting of frank gallbladder necrosis. The patient recovered well after a short post-operative stay. This report is intended to illustrate an unusual presentation of acute, gangrenous cholecystitis with herniation through the ventral abdominal wall.

## INTRODUCTION

Acute cholecystitis is a common medical diagnosis, with an estimated 120 000 Americans receiving treatment annually [1]. The majority of these patients will undergo cholecystectomy or surgical removal of the gallbladder, and while anatomic variability is inherent with any surgical procedure, the majority of cases are considered to be uncomplicated. Ventral incisional hernias are also a common complication of abdominal surgery with an estimated 2–20% incidence depending on the specific surgery performed [2]. While these two diagnoses are prevalent within the general population, only rarely have reports of gallbladder herniation been known to occur [3]. The presence of a gallbladder containing hernia is important because it impacts not only a surgeon’s approach to the procedure but also adds additional considerations to minimizing the risk of future visceral hernias. This report will cover the case of a patient who presented with a gallbladder containing ventral hernia and the discussion and operative decision making by the treatment team.

## CASE REPORT

**Figure 1:**
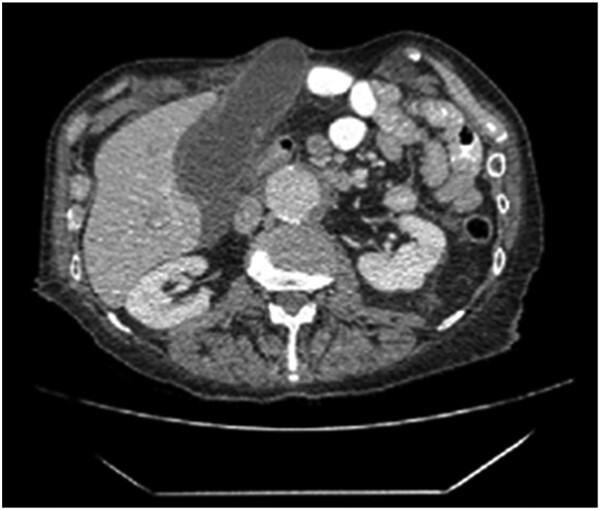
The axial section of the abdomen/pelvis CT scan illustrating our patient’s incisional hernia containing his gallbladder.

**Figure 2:**
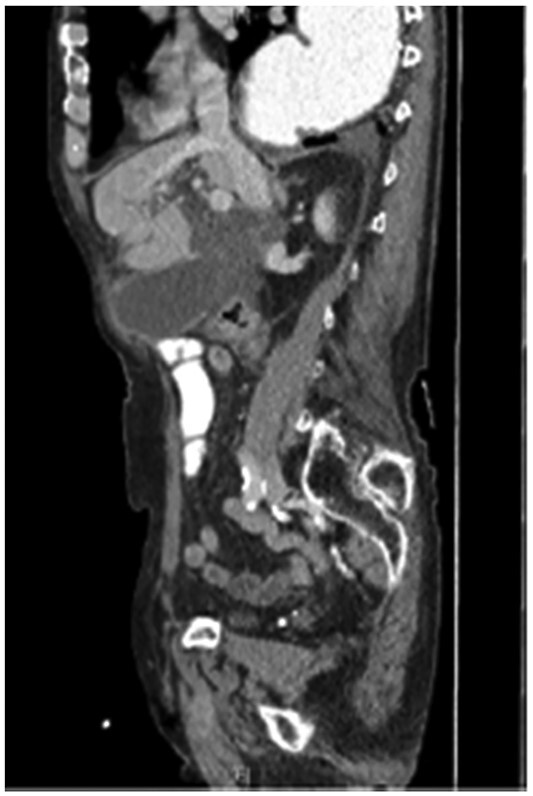
The coronal section of the abdomen/pelvis CT scan illustrating our patient’s incisional hernia containing his gallbladder.

Seventy-six-year-old male with a past medical history of COPD and esophageal cancer status post gastric pull-through procedure 25 years prior, presented to the Emergency Department with a chief complaint of sudden onset epigastric pain that began 24 hours prior while at rest. The pain progressively worsened and radiated to both his right upper quadrant and back with associated chills and nausea/vomiting. He was found to be febrile (38.1C) with a leukocytosis (18.77), hyperbilirubinemia (2.8), transaminitis (418/196) and elevated alkaline phosphatase (168) and lipase (>2250). A CT abdomen/pelvis with IV contrast demonstrated a thickened gallbladder wall, pericholecystic fluid and associated edema of the pancreas consistent with acute cholecystitis and pancreatitis. While the CT showed postoperative changes given his history of esophagectomy with gastric conduit, multiple unique anatomic abnormalities were also noted, including the presence of the gallbladder through a ventral hernia to where it was flush with the skin/subcutaneous tissue of the anterior abdomen (Figs 1 and 2). By hospital day 2, the patient’s lab values were downtrending, consistent with a transient common bile duct stone. Follow-up MRCP confirmed the diagnosis of acute calculous cholecystitis with obstruction of the cystic duct and patency of the common bile and pancreatic ducts. The decision was made to take the patient to the operating room for cholecystectomy with cholangiography. In the operating room, a right subcostal incision was made using electrocautery with a hernia sac encountered directly deep to the subcutaneous tissue. The peritoneum of the hernia was opened and the tip of the gallbladder was immediately encountered. On gross examination, necrosis was noted with evidence of microperforation into the hepatic plate, though no gross contamination was present. The gallbladder was dissected off the hepatic plate, the cystic artery was identified and ligated and stones palpable within the cystic duct were milked retrograde prior to ligation of the cystic duct. The gallbladder was removed and sent for definitive pathologic review. The hernia sac and skin were reapproximated in a multi-layered closure but the fascial edges were not amenable to primary closure secondary to both chronicity and size of the incisional hernia. The patient tolerated the procedure well and was able to discharge home on hospital day 5 (Post-operative day 3). Final pathology was consistent with acute calculous gangrenous cholecystitis. The patient was seen for follow-up 1 month post-operatively and was doing well with no complaints or return of symptoms.

## DISCUSSION

Gallbladder-containing hernias are rarely documented with only a small number of cases reports available in the literature. A literature review of gallbladder hernias by To *et al.*, found that of the 10 cases reported, three were within incisional hernias, with the remaining involving epigastric, parastomal and spontaneous ventral hernias [3]. The majority of these cases occurred in older aged individuals with a history of prior abdominal surgery, both of which are consistent with our patient’s presentation. Part of this is believed to be related to disruption of the patient’s normal anatomy, as well as the natural course of elongation of the gallbladder mesentery with age, making it more mobile and at risk for herniation in the right clinical context [3]. Our patient had a remote surgical history of an open esophagectomy with gastric pull-through procedure, an operation that often involves widening of the hiatus and extensive conduit mobilization [4]. It is likely that a combination of this prior mobilization and severe fascial retraction with resultant ventral hernia, contributed to the ability of the gallbladder to herniate as it did. Though it was not visualized on imaging, this patient’s gallbladder was found to be perforated into the cystic plate. We believe that operative intervention was necessary, as the patient would have been at risk for intra-abdominal perforation and resultant worsening sepsis if managed nonoperatively. All of the case reports of gallbladder herniations we reviewed were addressed surgically with open laparotomies except one case of herniation through a spontaneous ventral defect that was repaired laparoscopically [5]. In our case, an open approach allowed for adequate exposure of the hernia sac and neck, with reduced risk of intraperitoneal contamination given the setting of tissue necrosis. The decision to forgo repair of the ventral incisional hernia at the time of index operation was discussed in depth amongst our colleagues perioperatively. Permanent mesh placement is associated with the lowest rates of hernia recurrence; however, the risk of mesh infection leading to increased postoperative morbidity and subsequent operations when placed in a contaminated field is significant [6]. Complications including hernia recurrence and the need for mesh explant are both increased when permanent mesh is used for hernia repair during the same operation as a concomitant procedure at the same site [7]. Biologic mesh was also discussed but not pursued for this repair option as biologic mesh used in large ventral hernias have a higher rate of recurrence as compared to synthetic mesh [8]. As our patient had no history of hernia incarceration or strangulation, we believed it was safe to postpone definitive hernia repair. We recommended our patient follow-up for an elective repair with the goal of minimizing future risk of organ herniation, physical deformity and potential for impending hernia accident.

## AUTHORS’ CONTRIBUTIONS

All authors were involved in the operation and treatment of this patient. T.B. and W.C. prepared the manuscript and literature search. G.Q. gave the final approval of the version to be published. All authors read and approved the final manuscript.

## CONSENT FOR PUBLICATION

Written informed consent was obtained from the patient for publication of this case report. A copy of the written consent is available for review from the corresponding author of this manuscript upon request.

## CONFLICT OF INTEREST STATEMENT

The authors declare that they have no competing interests.

## FUNDING

None.
